# A Three-Stemmed mRNA Pseudoknot in the SARS Coronavirus Frameshift Signal

**DOI:** 10.1371/journal.pbio.0030172

**Published:** 2005-05-17

**Authors:** Ewan P Plant, Gabriela C Pérez-Alvarado, Jonathan L Jacobs, Bani Mukhopadhyay, Mirko Hennig, Jonathan D Dinman

**Affiliations:** **1**Department of Cell Biology and Molecular Genetics, University of MarylandCollege Park, MarylandUnited States of America; **2**Department of Molecular Biology and the Skaggs Institute for Chemical Biology, The Scripps Research InstituteLa Jolla, CaliforniaUnited States of America; University of WisconsinUnited States of America

## Abstract

A wide range of RNA viruses use programmed −1 ribosomal frameshifting for the production of viral fusion proteins. Inspection of the overlap regions between ORF1a and ORF1b of the SARS-CoV genome revealed that, similar to all coronaviruses, a programmed −1 ribosomal frameshift could be used by the virus to produce a fusion protein. Computational analyses of the frameshift signal predicted the presence of an mRNA pseudoknot containing three double-stranded RNA stem structures rather than two. Phylogenetic analyses showed the conservation of potential three-stemmed pseudoknots in the frameshift signals of all other coronaviruses in the GenBank database. Though the presence of the three-stemmed structure is supported by nuclease mapping and two-dimensional nuclear magnetic resonance studies, our findings suggest that interactions between the stem structures may result in local distortions in the A-form RNA. These distortions are particularly evident in the vicinity of predicted A-bulges in stems 2 and 3. In vitro and in vivo frameshifting assays showed that the SARS-CoV frameshift signal is functionally similar to other viral frameshift signals: it promotes efficient frameshifting in all of the standard assay systems, and it is sensitive to a drug and a genetic mutation that are known to affect frameshifting efficiency of a yeast virus. Mutagenesis studies reveal that both the specific sequences and structures of stems 2 and 3 are important for efficient frameshifting. We have identified a new RNA structural motif that is capable of promoting efficient programmed ribosomal frameshifting. The high degree of conservation of three-stemmed mRNA pseudoknot structures among the coronaviruses suggests that this presents a novel target for antiviral therapeutics.

## Abstract 2

A new structural and conserved element is identified within the SARS virus genome. The element is important for gene expression, and might be a useful target for antiviral drugs.

## Introduction

Severe acute respiratory syndrome (SARS) first appeared in Guangdong Province, China, late in 2002. Its rapid transmission and high rates of mortality and morbidity resulted in a significant threat to global health by the spring of 2003, and the epidemic had a significant effect on the public health and economies of locales affected by SARS outbreaks. The rapid response of the World Health Organization is credited with containing this contagion by late June 2003, and only a few cases were reported during the winter cold season of 2003–2004. The severity of this crisis mobilized the scientific community as well: by March 24, 2003, scientists at the Centers for Disease Control and Prevention and in Hong Kong had announced that a new coronavirus had been isolated from patients with SARS (reviewed in [1]). The sequences from two isolates of SARS-CoV were published simultaneously on May 1, 2003 [2,3].

Coronaviruses are enveloped animal viruses that cause respiratory and enteric diseases. Analysis of the SARS-CoV genome revealed that, similar to all coronaviruses, the 70% (approximately) at the 5′ end of its large single (+) stranded RNA genome consists of two sizable genes called ORF1a and ORF1b. The 3′ ORF1b overlaps, and is out of frame with, its 5′ neighbor, ORF1a, and similar to other coronaviruses, a programmed −1 ribosomal frameshift (−1 PRF) was posited to be used by the virus to produce an ORF1a/1b fusion protein [2]. A wide range of RNA viruses use −1 PRF for the production of viral fusion protein (reviewed in [4–6]). In many such cases, e.g., the Retroviridae and Totiviridae, the efficiency of ribosomal frameshifting determines the stoichiometric ratio between structural and enzymatic proteins available for viral particle assembly, and even small changes in frameshift frequencies can have profound negative effects on virus propagation, thus targeting −1 PRF for antiviral therapies (reviewed in [7]). It has been shown that the SARS-Cov −1 PRF signal is able to promote efficient frameshifting in a rabbit reticulcyte system, and the −1 PRF signal reported in that publication consisted of a typical heptameric “slippery site” (UUU AAAC), a 5-nt spacer, and a typical H-form mRNA pseudoknot containing two double-stranded RNA stems and two single-stranded loops [8]. Two very recently published papers have suggested that the SARS-CoV mRNA pseudoknot may contain a third stem-loop structure [9, 10]. In this work, we present computational, comparative genomic, molecular, biophysical, and genetic evidence demonstrating that the SARS-CoV frameshift signal includes a new type of highly ordered three-stemmed mRNA pseudoknot that likely contains a large number of noncanonical base interactions. Although total deletion of the third stem does not significantly alter frameshifting efficiency, its disruption significantly inhibits this process. The fact that this general structure appears to be conserved among the coronaviruses raises questions regarding its biological function.

## Results

### Computational Analysis of the SARS-CoV Frameshift Signal Suggests the Presence of a Three-Helix-Containing RNA Pseudoknot

**Figure 1:**
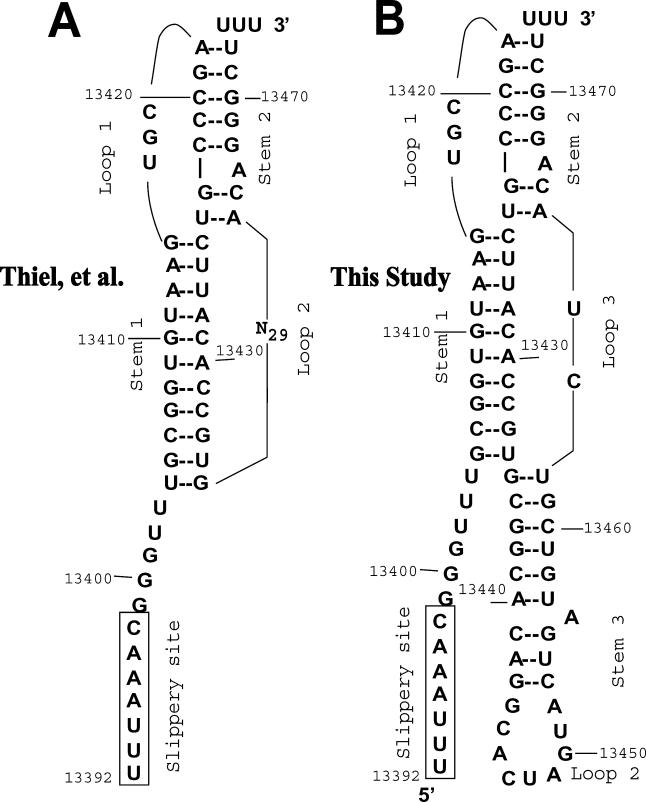
Different Representations of the SARS-CoV Frameshift Signal (A) Two-stemmed H-type mRNA pseudoknot proposed by Thiel et al. [8]. (B) Three-stemmed mRNA pseudoknot structure investigated in this study.

−1 PRF signals typically have a tripartite organization. From 5′ to 3′, these are composed of a heptameric “slippery site,” a “spacer” region, and a stable mRNA secondary structure, typically an mRNA pseudoknot (reviewed in [11]). A previous analysis of the SARS-CoV −1 PRF signal demonstrated that a sequence spanning nucleotide positions 13392–13472 satisfied these three requirements and was able to promote efficient −1 PRF in rabbit reticulocyte lysates [8]. The −1 PRF signal presented in that study contained a typical mRNA pseudoknot composed of two double-helical, Watson–Crick basepaired stems connected by two single-stranded loops (Figure 1A).

The presence of a long, 29-nt loop 2 seemed to be unusual, prompting us to subject the sequence from positions 13392–13472 to additional computational analyses in an effort to further define the structure of this mRNA pseudoknot. The nucleotide sequence suspected of featuring a −1 PRF signal between ORF1a and ORF1b was scanned by RNAMotif [12], using a pattern-based description capable of finding common −1 PRF signals in other RNA viruses. As expected, a so-called slippery site (UUU AAAC) and a large H-type pseudoknot were identified—the two primary stimulating elements required for efficient ribosomal slippage. This analysis was coupled with Pknots [13], a software package that predicts the most thermodynamically stable structure for a given RNA sequence. The predicted structure for the SARS-CoV frameshift signal was extremely stable, with a calculated minimum free energy (MFE) of −26.68 kcal/mol. The surprising result was that the 29-nt sequence designated loop 2 by Thiel et al. [8] was predicted to form a third helix, nested within the sequences defined by stems 1 and 2 (Figure 1B). Though a small, internally nested third helix (helix-3) has been shown to be present in the HIV-1 group O frameshift signal [14], such an extensive basepairing pattern has not to our knowledge been heretofore demonstrated for any other viral frameshift signal. To determine the statistical significance of this finding, a distribution of MFE values taken from 500 randomly shuffled SARS-CoV frameshift signals was created. Each of the randomly shuffled sequences was folded using Pknots with the same parameters. The resulting normal distribution had mean MFE of −21.12 kcal/mol (standard deviation = 2.67, 500×), revealing that the predicted three-stemmed pseudoknot structure of the native sequence is highly significant with a *z*-score of −2.05 and a *p* value of 0.02 (one-tailed Student's *t*-test).

### Phylogenetic Conservation of Predicted Three-Stemmed mRNA Pseudoknots in Coronaviruses

**Figure 2:**
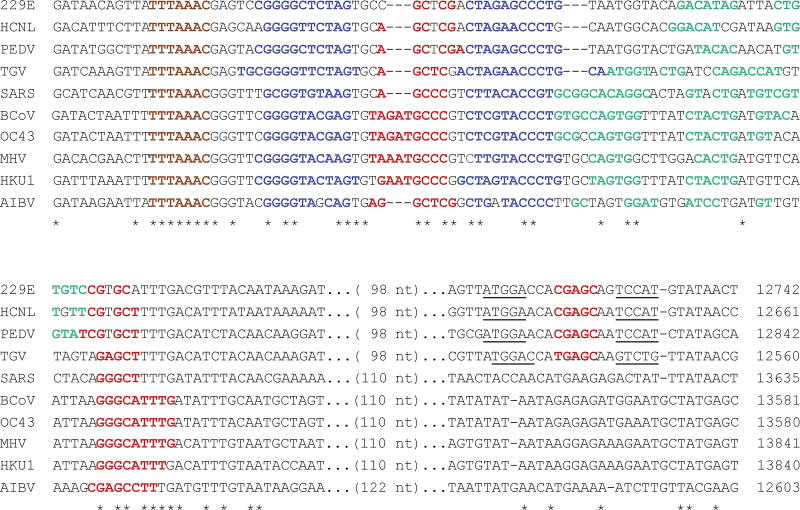
Multiple Sequence Alignment of the SARS-CoV −1 PRF with Nine Homologous Signals Found in Other Coronavirus Genomes AIBV, avian infectious bronchitis virus; BCoV, bovine coronavirus; HCoV-229E, human coronavirus 229E; HCoV-HKU1; HCoV-NL63, human coronavirus NL63; HCoV-OC43, human coronavirus OC43; MHV, murine hepatitis virus; PEDV, porcine epidemic diarrhea virus; SARS, SARS coronavirus; TGV, transmissible gastroenteritis virus. Heptameric slippery sites are indicated in brown; dashes indicate gaps in the sequence alignments; basepairing positions involved in the consensus first, second, and third helices are denoted by blue, red, and green nucleotides, respectively. Downstream regions homologous to the kissing loop known to promote frameshifting in HCoV-229E [16,17]. HCoV-229R, HCoV-NL, PEDV, and TGV are also highlighted in red with the flanking stem-forming sequences underlined. Asterisks indicate perfectly conserved positions in primary sequence.

**Figure 3:**
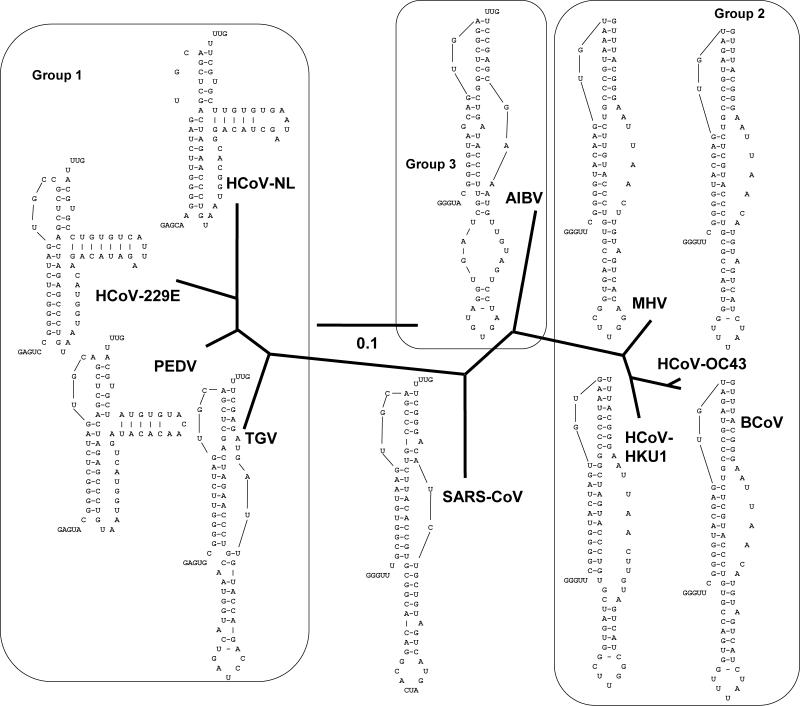
Phylogenetic Analyses of Coronavirus −1 PRF Signals Unrooted tree constructed based on the multiple sequence alignment from Figure 2.

To address the question of whether the potential to form a three-stemmed mRNA pseudoknot is unique to the SARS-CoV, we searched for such structures in all of the known viral −1 PRF signals listed in the RECODE 2003 database [15], as well as the putative frameshift signals in all of the sequenced members of the Order Nidovirales (including coronaviruses and arteriviruses). The SARS-CoV frameshift signal itself is homologous to all of the nine other frameshift signals for coronaviruses whose genomes have been fully sequenced. A multiple sequence alignment of the ten coronavirus frameshift signals is presented in Figure 2. This shows that both stems 1 and 2 are highly conserved, with a strong conservation of base complementation in the cores of both stems 1 and 2 (blue and red sequences, respectively). This analysis also shows all of the coronavirus frameshift signals have the potential to form a third helix, although the structures and sequences are less well conserved (Figure 2, in green). In addition, the potential of sequences located approximately 200 nt downstream of the slippery site to form long-range “kissing loop” interactions with the 5′ half of stem 2 was previously noted for HCoV-229E [16] and TEGV [17]. This property was only conserved among all of the group 2 coronaviruses, not in any of the others (see Figure 2). The potential significance of this observation is discussed below. A phylogenetic tree of the −1 PRF signals constructed from the multiple sequence alignment is presented in Figure 3. As expected, the group 1 and group 2 coronaviruses cluster together, and neither the SARS-CoV nor the avian infectious bronchitis virus (AIBV) frameshift signals cluster with either group. Of particular interest, however, is that very similar mRNA pseudoknot structures are predicted to occur within groups—but not between them.

### Nuclease Mapping of the SARS-CoV Frameshift Signal Is Consistent with the Presence of a Complex, Three-Helix-Containing RNA Pseudoknot Structure

**Figure 4:**
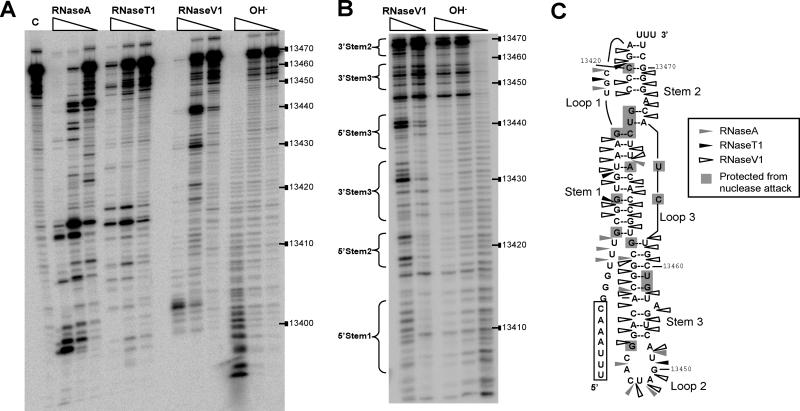
Secondary Structure Mapping of the SARS-CoV Frameshift Signal (A and B) The results of nuclease cleavage of RNA from nucleotides 13400–13470 of SARS-CoV. RNAs were 5′ end labeled with ^32^P and subjected to enzymatic digestion, as described in Materials and Methods. The three different concentrations of each nuclease are indicated by the triangles are described in Materials and Methods. C denotes undigested control, and OH^−^ denotes hydrolysis ladders. (C) Interpretation of nuclease digestion analyses mapped onto the proposed secondary structure of the SARS-CoV frameshift signal. Nuclease cleavage sites, proposed basepairs, and specific bases protected from nuclease attack are indicated.

In light of the computational findings, we conducted biochemical analyses of the SARS-CoV frameshift signal using a [^32^P] 5′ end labeled SP6 RNA polymerase product spanning nucleotides 13399–13475. RNase A cleaves preferentially at single-stranded pyrimidine bases, RNase T1 cuts at single-stranded guanosine residues, and RNase V1 cleaves double-stranded RNA. We also examined alkaline hydrolysis cleavage patterns at low concentrations of sodium hydroxide to identify exposed phosphodiester bonds. Representative autoradiograms of the reactions are shown in Figure 4A and 4B, and the predicted cleavage patterns mapped onto the pseudoknot structure are shown in Figure 4C.

The nuclease mapping data are generally consistent with the computational predictions, showing double-stranded regions corresponding to all three stems. Some notable deviations from the predicted structure were observed, however. These fell into three general classes. One class consisted of distortions in predicted helical A-RNA structures, typified by bases that were equally digested by both single- and double-strand-specific nucleases and by nearby bases that were refractory to nuclease attack. These clustered in the middle of stem 1 (13406–13410 and 13427), near the middle of stem 2 (13419–13420), and in the middle of stem 3 (13436–13439 and 13458–13461). Another major group consisted of bases located in regions predicted to link the three stems that were completely protected from nuclease attack. Specifically, these were G13405 and G13435 at the stem 1/stem 3 junction, G13414 and G13423–C13425 at the stem 1/stem 2 junction, and C13463 and U13464, which link stem 2 with stem 3. We also observed enhanced susceptibility of the three pyrimidines in the predicted loop 2 region (C13447, U13448, and U13451) to attack by both single- and double-strand-specific endonucleases, suggesting that this region is structurally dynamic under the conditions assayed. The ability of the bulged adenosine residue at position 13467 to be recognized by RNaseV1 demonstrates that it is involved in a basepairing interaction, whereas the opposite pertains with regard to A13446, A13452, and A13456. The three bases at the 5′ and 3′ terminal ends of the molecule could not be meaningfully resolved.

### Two-Dimensional Nuclear Magnetic Resonance Analysis Confirms the Presence of Three Stems

**Figure 5:**
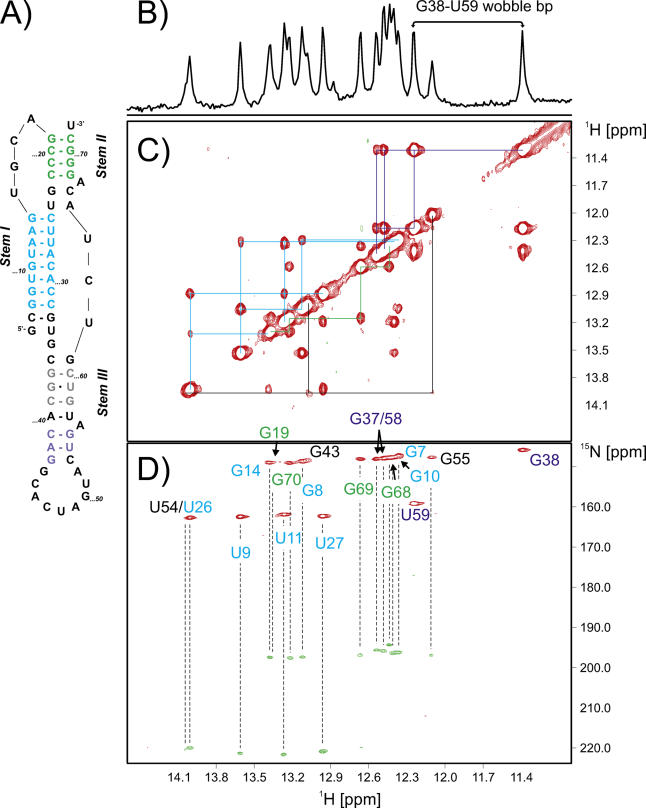
NMR Data Were Collected at 25 °C at a Proton Resonance Frequency of 900 MHz (A) Secondary structure of the SARS-CoV frameshift pseudoknot (residues 13405–13472). Different color coding was used to denote basepaired regions in stems 1 (cyan), 2 (green), and 3 (grey and blue). Only the last two digits of the wild-type sequence numbering are used for clarity. (B) Imino region of a one-dimensional jump-return echo spectrum of SARS-CoV pseudoknot. (C) Portion of a 2D ^1^H,^1^H-NOESY. Sequential imino-imino proton NOE assignment paths are shown by different colors for stem 1 (cyan), stem 2 (green), and stem 3 (black and blue). (D) 2D Quantitative *J*(N,N) HNN-COSY spectrum showing interstrand ^1^H3–^15^N3(U) to ^15^N1(A) and ^1^H1–^15^N1(G) to ^15^N3(C) correlations. Data were collected on a uniformly ^13^C/^15^N-labeled sample. Red peaks correspond to diagonal resonances and are labeled with assignment information for the basepaired stem regions matching the color coding in (C). Green cross peaks are caused by scalar cross hydrogen bond ^2h^ *J*(N,N) couplings detected using a defocusing delay of 36 ms. Carrier positions were on water for ^1^H and 185 ppm.

Given the ambiguity of the nuclease mapping, homo- and heteronuclear two-dimensional (2D) nuclear magnetic resonance (NMR) experiments were used to confirm the predicted basepairing interactions of the three stems. The presence of 21 hydrogen-bonded guanine and uracil residues for the sequence including residues 13405–13472 of the SARS-CoV genome (Figure 5A) was evident from the imino region observed in the 2D ^1^H,^1^H-NOESY, ^15^N-HMQC, and quantitative *J*(N,N) HNN-COSY data. In this study, we have obtained sequential imino ^1^H and ^15^N assignments for the A-form helices of the frameshifting SARS pseudoknot, using a combination of information from ^1^H,^1^H-NOE and ^1^H,^15^N-HMQC spectra. The latter experiment distinguishes between uridine and guanosine iminos by the characteristic ^15^N chemical shift, whereas the NOESY yields sequential ^1^H,^1^H-NOEs connecting imino protons in helical stem regions.

Assignments were made for 8 bp in stem 1, 4 bp in stem 2, and 5 bp in stem 3 (Figure 5). As a result of fraying, the terminal basepairs of helical stem regions are not observed. Of those basepairs assigned, 16 are Watson–Crick-type basepairs and only one is a canonical wobble G:U basepair. This G:U basepair present in stem 3 can be inferred directly from the strong NOE correlation between the G38 and U59 imino protons (Figure 5C). The corresponding donor G38:N1 and U59:N3 imino nitrogens are evidently not engaged in G:C or U:A hydrogen bonds (Figure 5D).

The quantitative *J*(N,N) HNN-COSY contains a total of five correlations between the imino N3 nitrogens of uridines and the N1 nitrogens in adenines, indicative of canonical Watson–Crick-type basepairing interaction. A total of 11 correlations stemming from Watson–Crick G:C basepairs are observed between the imino N1 nitrogens of guanosines and the N3 nitrogens of cytidines. In summary, the complete sequential NOE walk connecting most of the basepaired imino protons unambiguously confirmed the presence of three stems corresponding to the secondary structure prediction shown (Figure 5A).

### The Predicted SARS-CoV Frameshift Signal Functions Like Other −1 PRF Signals

To address the question of whether the predicted SARS-CoV −1 PRF signal functions similarly to −1 PRF promoting elements from other viruses, this sequence was cloned into bicistronic dual luciferase reporter constructs designed to assay programmed ribosomal frameshifting using in vitro and in vivo systems [18,19]. As a minor modification, instead of using a simple readthrough construct as the zero-frame control, the corresponding control contained the SARS-CoV −1 PRF signal with one additional base inserted 3′ of the Renilla luciferase sequence and 5′ of the −1 PRF signal. The resulting zero-frame reporter places the firefly luciferase ORF in frame with Renilla and inactivates the −1 PRF signal by moving it out of frame with regard to elongating ribosomes, while controlling for ribosomes dislodged from the reporter mRNAs by the mRNA pseudoknot. This seemed to alleviate the large errors observed by other groups using similar methodology (e.g., [9]).

**Figure 6:**
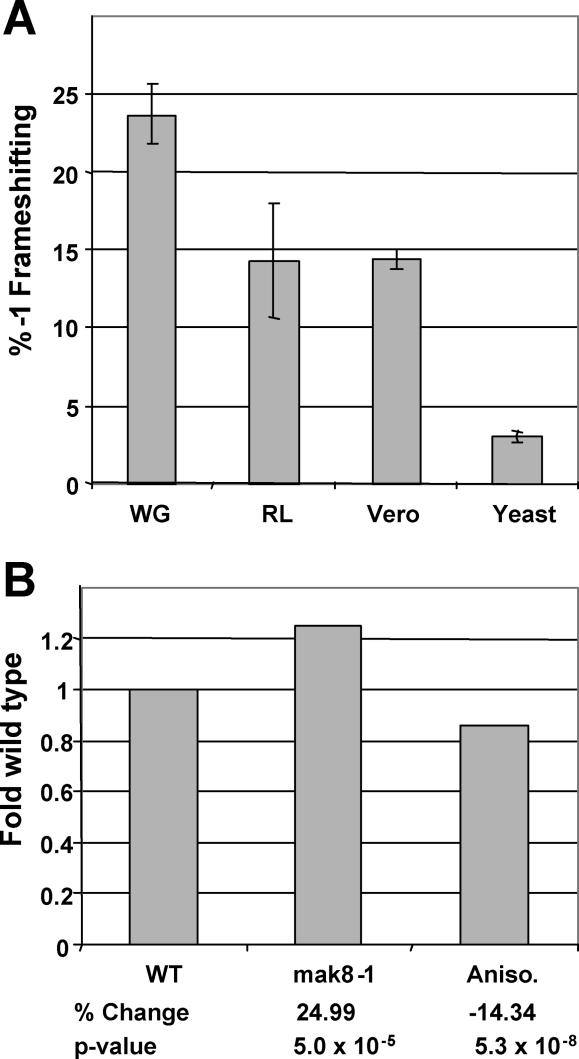
Functional Characterization of the SARS-CoV Frameshift Signal (A) The wild-type SARS-CoV frameshift signal promotes efficient frameshifting in vitro and in vivo. Programmed −1 ribosomal frameshifting was monitored in wheat germ and rabbit reticulocyte lysates in vitro, and in Vero epithelial cells and yeast in vivo, as described in Materials and Methods. Error bars denote the standard error. (B) SARS-CoV-directed −1 PRF was monitored in wild-type yeast cells with or without anisomycin (20 μg/ml), or in isogenic *RPL3* gene deletion cells expressing either the wild-type or *mak8–1* alleles of *RPL3* on an episomal plasmid [21]. Changes in −1 PRF efficiencies are shown as fold wild-type, and *p*-values are shown as described previously [25].

The ability of the SARS-CoV sequence to promote −1 PRF was assessed using two different in vitro and in vivo assay systems each. The results of these experiments are shown in Figure 6A. In vitro, the SARS-CoV sequence was able to promote efficient −1 PRF in both wheat germ protoplasts (23.7% ± 1.9%) and rabbit reticulocytes (14.3% ± 3.7%). In vivo, the sequence was able to promote efficient −1 PRF in the Vero epithelial cell line (14.4% ± 0.6%), a finding that is important in light of the fact that the SARS-CoV infects lung epithelial cells. The sequence also promoted efficient −1 PRF in yeast cells, suggesting that this frameshift signal might be amenable to the molecular genetic toolbox available in the yeast system. To test this hypothesis, we examined the effects of a drug (anisomycin) and of a host cell mutant *(mak8–1)* that were previously shown to specifically affect L-A virus–directed −1 PRF in yeast cells [20–22]. The results of these experiments show that, similar to their effects on L-A-promoted −1 PRF, anisomycin was able to inhibit SARS-CoV-directed −1 PRF (21% inhibition, *p* = 5.04 × 10^−8^), whereas −1 PRF was stimulated in cells harboring the *mak8–1* allele of *RPL3* (25% stimulation, *p* = 5.0 × 10^−5^) (Figure 6B). These findings show that the SARS-CoV frameshift signal is amenable to analysis by the full array of yeast-based genetic, pharmacological, and molecular tools that we and others have developed. Interestingly, the absolute values for frameshifting in yeast (2.99% ± 0.06%) were significantly less than those observed in the other systems (ranging from approximately 15% to 25%), suggestive of differences between fungal and metazoan ribosomes that might be pharmacologically exploited. This is discussed in greater detail below.

### Structural Requirements for Efficient SARS-CoV Frameshifting Activity

**Figure 7:**
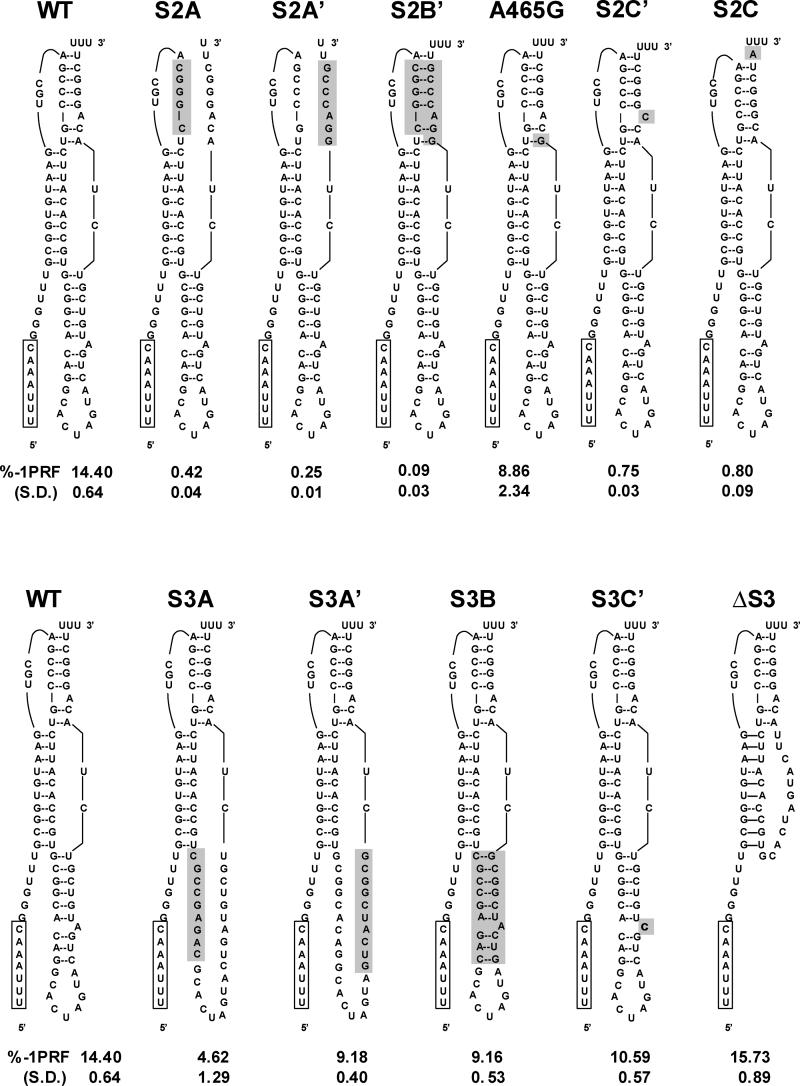
Molecular Genetic Analyses of Stems 1 and 3 Constructs used to examine the contributions of stem structures and bulged adenosine residues to programmed −1 ribosomal frameshifting are depicted. Shading is used to indicate mutagenized bases. Programmed −1 ribosomal frameshifting promoted by the wild-type SARS-CoV −1 PRF signal was monitored in Vero, as described in the Materials and Methods. Standard deviations (S.D.) are indicated for each sample, as previously described [25]. The S2 series (above) examines the roles of structures and bases in stem 2. The S3 series (below) examines the roles of structures and bases in stem 3.

Given that Vero cells more resemble the natural host of SARS-CoV than do yeast, a series of mutants of the SARS-CoV frameshift signal were developed to functionally dissect the mRNA pseudoknot in this cell type. Typically, mutagenesis experiments are constructed so as to change one or another side of a stem to disrupt basepairing, and then to combine the two mutants to re-form the stem (e.g., see [23,24]). The series of mutants that were created by oligonucleotide site-directed mutagenesis to address this question is shown in Figure 7. The S2 series of mutants were designed to examine the general requirement for stem 2, and the specific contribution of the bulged adenosine residue at position 13467 was designed to stimulate efficient −1 PRF. Similarly, the S3 mutant series were designed to examine the general requirement for stem 3, as well as the specific contribution of the bulged adenosine at position 13456. The complete data for these experiments, as formatted according to [25], are presented in Dataset S1.

Six different stem 2 mutants were assayed for their ability to promote efficient −1 PRF (Figure 7). Not surprisingly, disruption of stem 2 (S2A and S2A′) precluded efficient −1 PRF. Unexpectedly, however, compensatory mutations that should promote re-formation of the basic stem 2 structure (S2B′) did not restore wild-type levels of frameshifting, suggesting the involvement of a primary mRNA sequence in this region in stimulating −1 PRF. However, the adenosine base at position 13465 in this construct had to be replaced with a guanosine to avoid creating a −1 frame termination codon. Though this substitution retains the potential to basepair with U13424, it is possible that the identity of the base at this position is critical. To examine this parameter, the base at this position was changed to guanosine in the context of an otherwise wild-type −1 PRF signal (A13465G). Though this mutation did not abrogate efficient −1 PRF, frameshifting efficiency was decreased by approximately 38% (*p* = 1.7 × 10^−6^). This result suggests that though this mutation was not the main cause of the dramatic reduction in −1 PRF observed with S2B′, the identity of the base at this position is important for maximizing −1 PRF efficiency. These observations are consistent with the hypothesis that both the general structure and base-specific sequence of stem 2 are required for efficient −1 PRF.

Bulged adenosine residues are known to stimulate assembly of higher-order RNA structures by helping to link helices together [26]. Two constructs were assayed to examine the requirement of the bulged residue at position 13467 for efficient −1 PRF. In mutant S2C′, A13467 was substituted with cytosine, whereas in construct S2C, the adenosine at position 13467 was removed from the middle of stem 2 and repositioned six bases downstream to maintain translational reading frame. Either replacing the A-bulge with cytosine (S2C′) or deleting it entirely (S2C) dramatically reduced frameshifting in Vero cells (>94%, *p* < 3.3 × 10^−16^), repressing −1 PRF to a similar extent as the mutants S2A, S2A′, and S2B′.

Similar to the approach described above, five mutants were constructed to investigate stem 3 (Figure 7). Constructs S3A, S3A′, S3B, and S3C′ were directed toward addressing the function of stem 3: in S3A, the guanine and cytosine residues in the 5′ half of stem 3 were mutated to cytosine and guanine, respectively, disrupting stem 3; the opposing mutations were made in the 5′ half of stem 3 in S3A′; and S3B harbored the compensatory mutations to allow re-formation of stem 3. Frameshifting with S3A was reduced by 68% (*p* = 2.61 × 10^−24^), and −1 PRF was significantly, although less dramatically, reduced in S3A′ (36% of wild-type, *p* < 1.07 × 10^−19^). Similar to the effects observed in stem 2, the presence of compensatory mutations in construct S3B did not rescue −1 PRF efficiency to near wild-type levels, again suggesting that both the general structure of stem 3 and specific sequences within it are required for maximal stimulation of −1 PRF.

Similar to stem 2, a bulged adenosine is predicted in stem 3 at position 13456, and the phylogenetic analysis showed that this base was conserved among all of the coronaviruses. Substitution of this base to cytosine (S3C′) promoted a moderate but significant reduction in −1 PRF (26% inhibition, *p* = 2.56 × 10^−11^). In addition, as no significant internal nested stems have been observed in other viral frameshift pseudoknots, and because deletion of sequence corresponding to this region did not dramatically affect −1 PRF in AIBV [27], the entire stem 3–forming region was deleted in construct ΔS3 to create a more typical two-stemmed H-type RNA pseudoknot. In Vero cells, this smaller pseudoknot, lacking the third nested helix, actually promoted a modest increase in frameshifting (9.2%, *p* = 2.15 × 10^−3^), demonstrating that stem 3 is not critical for promoting efficient −1 PRF per se.

An analogous series of constructs were also assayed in yeast (data not shown). In general, the trends were similar, though the actual baseline frameshifting efficiencies were lower. For example, in mutants S2A, S2A′, and S2B′, frameshifting was equally reduced by 85%–90%; the S3A and S3A′ mutations also resulted in moderate (35%–89%) decreases in yeast, and deletion of stem 3 (ΔS3) also presented a slight increase in −1 PRF (35%) in yeast cells. There were some notable contrasts, however: though the S2C′ and S2C constructs dramatically reduced frameshifting in Vero cells (95%), they only reduced −1 PRF by approximately 25%–33% in yeast. More strikingly, some of the mutations that resulted in a 25%–30% decrease in −1 PRF in Vero cells (A13465G, S3B, and S3C′) did not affect the overall rate of −1 PRF in yeast at all. The potential significance of these findings is discussed below.

## Discussion

Though the first descriptions of the mRNA secondary structure stimulating −1 PRF [28] and of an RNA pseudoknot [29] were serendipitously published back to back in 1985, the two concepts were only functionally linked together 1 y later in studies of a coronavirus, AIBV [30]. Here, the coronaviruses have again revealed a new twist on mRNA pseudoknots and −1 PRF. The phylogenetic comparisons presented in this study reveal that stem 1 lengths and G:C compositions are highly conserved in all ten coronavirus sequences analyzed. Their relatively long G:C-rich composition presumably contributes significantly to the stability of these structures. In contrast, stem 2 structures are predicted to vary significantly between the different coronavirus groups. Specifically, the group 2 coronaviruses (HCoV-043C, HCoV-HKU1, BCoV, and MHV) have the longest and most stable predicted stem 2 structures, whereas the stem 2 regions of the group 1 coronaviruses (TGV, PDEV, HCoV-NL, and HCoV-229E) are anticipated to be the least stable. The stem 2 regions of SARS-CoV and AIBV appear to be intermediate between these two. Although the sequences in the stem 3/loop 3 region are not well conserved, a third stem independently predicted in the SARS-CoV −1 PRF signal [10] has been demonstrated in this work, and we predict third stems in other coronavirus frameshift signals. Similar structures are generally predicted to be able to form within groups.

Specifically, loop 3 is predicted to be long and positioned between stems 3 and 2 in the group 2 coronaviruses. In contrast, the group 1 viruses contain little or no loop 3 but, rather, have an extended loop 2 positioned between stems 1 and 3. The notable exception is TGV, in which the relative structure and orientation of stem 3 and loop 3 more resembles those observed in SARS-CoV and AIBV.

Structurally, the nuclease analyses, showing distortions of the regular helical structures in the stems, protection of specific bases from nuclease attack, and the apparent involvement of bases in loop 3 and of A13467 in basepairing interactions, suggest that the three stems fold back on one another to form a complex, globular RNA structure. Long-range interaction anchors mediated by adenosine residues such as those at positions 13456 (stem 3) and 13467 (stem 2), making contact with the shallow minor grooves of two stacked basepairs of A-form helical stems, are a recurring theme in RNA structural biology. For example, the crystal structure of the ribosome reveals that RNA has a remarkable propensity for contributing adenine bases to such A-minor interactions [31], thereby stimulating the assembly of higher-order RNA structures [26]. Mounting evidence suggests that stimulation of −1 PRF by mRNA pseudoknots requires specific noncanonical basepairing between helical stems and pseudoknot loop regions to set specifically required frameshift efficiencies. The structures of the few frameshift-promoting pseudoknots that have been determined at the atomic level are revealing that a large range of higher-order noncovalent interactions serve to promote stable, novel structures [32–36]. It is clear that the three-helix-containing mRNA pseudoknot described here represents a novel global architecture stimulating ribosomal frameshifting, and possibly a source of new structural motifs in the coming future. Experiments are currently underway to define this structure at the atomic level, using high-resolution NMR techniques. Elucidation of this novel mRNA structure will be of great utility in the rational development of therapeutic agents designed to interfere with SARS-CoV programmed −1 ribosomal frameshifting, and in furthering our understanding of how different pseudoknots stimulate translational recoding.

Molecular genetic analysis of stem 2 of the SARS-CoV pseudoknot, demonstrating that frameshifting was reduced in all cases, including our attempts to make complementary mutations, indicates that primary sequences as well as structures are important for maximal frameshifting. Possible reasons for the observed sequence specificity could include aberrant folding or disruption of an essential interaction required for formation of the complex tertiary mRNA structure. For example, the findings that changing the identities of the bulged adenosine residues in stems 2 and 3 from adenosine to cytosine (S2C′ and S3C′), or deleting the bulge in stem 2 altogether (S2C), abrogated the stimulatory effects of the pseudoknot support the notion that this structural property of bulged adenosine residues is functionally important in this context. In addition, water-nucleobase “stacking” in the form of H-π and lone pair-π interactions have been demonstrated at the junctions between the stems in the BWYV pseudoknot [34]. The corresponding regions of the wild-type SARS-CoV pseudoknot were refractory to nuclease attack and were disrupted in S2A, S2A′, S2B′, S3, S3A′, and S3B, possibly explaining the effects of all of these mutants on frameshifting. The changes made in S2C and S2C′ are also adjacent to this region, and inhibition of frameshifting is nearly as dramatic as with the S2A, S2A′, and S2B′ mutants.

Though alterations to stem 3 significantly reduced frameshifting levels, these effects were one to two orders of magnitude less than analogous mutations of stem 2. This is supported by the observation in another study that alteration of the sequence in stem 3 also promoted decreases in −1 PRF [9]. Further, complete deletion of stem 3 had only a minimal effect on frameshifting efficiency—an observation consistent with studies in AIBV, in which deletion of all but 5 nt between stems 1 and 2 did not significantly alter −1 PRF [27,37]. These findings demonstrate that the presence of stem 3 is not required for efficient frameshifting per se. However, its high degree of conservation among the coronaviruses and its location in the frameshift signal suggest that it plays a more complex role in programmed −1 ribosomal frameshifting as it relates to the viral life-cycle. A similar conclusion was drawn by the authors of another independent study that was performed concurrently with ours and that was published while this manuscript was under review [9].

If stem 3 is not required to promote efficient frameshifting, why then has it been so highly conserved among the coronaviruses? It may be that frameshifting levels in coronaviruses need to be regulated in a manner not supported by a two-stem pseudoknot. For example, the frameshift signal marks the boundary between proteins required during the immediate early phase of infection (e.g., ORF1a-encoded proteases used to prepare the cell for virus production) and those required for intermediate functions in the viral life cycle (i.e., ORF1b-encoded RNA-dependent RNA polymerase and helicase used in transcription of subgenomic mRNAs, [−] strand synthesis, and genome replication). One of the fundamental problems of in the biology of (+) RNA viruses regards the switch between translation and replication. An elegant model proposes that the −1 ribosomal frameshift in barley yellow dwarf virus plays a central role in remodeling the (+) strand from translation competent to replication competent: frameshifting enables synthesis of the replicase, which in turn is able to denature the frameshift-promoting *cis*-acting element, eventually clearing the (+) strand of ribosomes that could potentially block the replicase [23]. In coronaviruses, the idea of functional switching by RNA remodeling has been demonstrated for MHV [38], and similar functional elements are present in both SARS-CoV and BCoV [39].

In a previous study, frameshifting in HCoV-229E was shown to be stimulated by a short sequence approximately 200 nt downstream from the slippery site, and it was shown that efficient frameshifting is promoted by kissing-loop interactions [16]. A subsequent report also found this potential motif in the TGV genome [17]. The phylogenetic analysis presented here reveals the potential to form similar short imperfect stem 2 structures for the other two group 1 coronaviruses for which the sequence is known (HCoV-NL and PEDV). In contrast, similar interactions cannot be readily discerned in SARS-CoV, nor among the group 2 and group 3 coronaviruses. Nevertheless, the idea that viral sequences in the pseudoknot may interact either in *cis* with sequences on the (+) strand or in *trans* with either sequences in subgenomic mRNAs or on the (−) strand to modulate frameshifting remains an intriguing possibility.

A final finding of interest derives from the observed differences between yeast- and metazoan-derived frameshift assay systems. This represents a potentially exciting avenue of exploration, as it may be indicative of mRNA folding differences between the two systems or of differences in how yeast versus metazoan ribosomes interact with downstream mRNA structures. This could be a result of relative size differences in the ribosomes. Alternatively, the lower levels of frameshifting in yeast relative to wild-type in the Vero cells could reflect a higher sensitivity of these ribosomes to subtle changes in the frameshift signal. The normal levels of frameshifting in yeast promoted by the S3B and S3C′ mutants further support the notion that the reason for stem 3 may lie with some function other than programmed ribosomal frameshifting.

## Materials and Methods

### 

#### Computational analyses

The SARS-CoV −1 PRF signal was identified from the complete genome sequence, using a combined approach. First, a pattern matching descriptor of known −1 PRF signals was used in conjunction with RNAMotif [12] to identify the nucleotide sequence corresponding to the frameshift signal's slippery site. Second, Pknots [13] was employed to “fold” the sequence immediately downstream (3′) to the slippery site and to produce a predicted MFE value in kilocalories per mole for the sequence. The statistical significance of the predicted MFE value of the three-stemmed RNA pseudoknot was tested by generating 500 randomly shuffled sequences derived from the native sequence, refolding each of these, and calculating their MFE values using Pknots. This resulted in a normal distribution of MFE values, against which the native sequence could be compared and *z*-scores calculated. FASTA3 v3.4 [40] was used to initially identify sequences homologous to the SARS −1 PRF signal based on primary sequence similarity. The search space included 1,724 viral genome sequences downloaded using the National Center for Biotechnology Information's Entrez Taxonomy Browser [41]. The resulting pairwise alignments produced by FASTA3 were used to produce a multiple-sequence alignment using ClustalW v1.82 [42]. An unrooted phylogenetic tree was created from this alignment and visualized using Tree View v1.6.6 [43].

#### Strains, genetic methods, and programmed ribosomal frameshifting assays

Escherichia coli strain DH5α was used to amplify plasmids, and E. coli transformations were performed using the high-efficiency transformation method of Inoue et al. [44]. YPAD and a synthetic complete medium (H−) were used as described previously [45]. Yeast strain JD932 *(MATa ade2–1 trp1–1 ura3–1 leu2–3,112 his3–11,15 can1–100)* and the JD1228/JD1229 isogenic pairs in which the disrupted *RPL3/TCM1* allele is complemented with pRPL3 or pmak8–1 *(MATα ura3–52 lys2–801 trp1δ leu2^=^ his3 RPL3::HIS3)* [21] were used for in vivo measurements of −1 PRF. Yeast cells were transformed using the alkali cation method [46]. Dual luciferase assays for programmed ribosomal frameshifting in yeast were performed as previously described [19]. African green monkey Vero cells were cultured in DMEM with L-glutamine (BioWhittaker, Walkersville, Maine, United States) and 10% FBS at 37 °C in 5% CO_2_. Cells cultured without antibiotics were transformed with plasmid DNA, using Amaxa (Cologne, Germany) Nucleofector solution according to the manufacturer's instructions. Dual luciferase assays were performed the following day, using extracts from cells lysed with the Passive Lysis Buffer (Dual-Luciferase Reporter System, Promega, Fitchburg, Wisconsin, United States). Wheat germ and rabbit reticulocyte lysates from Ambion (Austin, Texas, United States) were used to monitor frameshifting in vitro, using synthetic mRNA transcripts (Ambion mMESSAGE mMACHINE transcription kit), generated with T7 polymerase either from plasmids that had been digested with SspI, Proteinase K treated, phenol/chloroform and chloroform extracted, and ammonium acetate precipitated, or from PCR amplicons encompassing the dual luciferase reporter cassettes. All assays were repeated until the data were normally distributed, enabling statistical analyses both within and between experiments [25]. At least three readings derived from lysates derived from a minimum of three different transfection plates were used.

#### Oligonucleotides, plasmid construction, and mutagenesis.

Oligonucleotides were synthesized and purified by IDT (Coralville, Iowa, United States). These are listed in Table 1. The SARS-sense and SARS-antisense oligonucleotides were annealed, gel purified, and ligated into BamHI- and SacI-digested p2luc [18], generating plasmid pJD435. The Renilla and firefly bicistronic elements were amplified by PCR using previously described primers [19], SpeI- and XhoI-digested, and cloned into p416ADH [47]. One additional base was introduced after the BamHI restriction site, using the Stratagene (La Jolla, California, United States) QuikChange XL kit to correct the reading frame. Sequence analysis revealed an additional point mutation in the firefly luciferase gene that was reverted by oligonucleotide site-directed mutagenesis. The resulting plasmid, pJD465, constituted the wild-type SARS-CoV −1 PRF yeast assay plasmid. A zero-frame control plasmid, pJD474, was constructed by adding one cytosine residue upstream of the BamHI restriction site of pJD465. Additional constructs with various mutations in the pseudoknot were made; the 5′ portion of stem 3 was changed from GCGGCACAG to CGCCGAGAC (pJD467, also known as S3A), and this was the template for mutagenesis to make the complementary mutation in the 3′ half of stem 3, CUGAUGUCGU to GUCU ACGGCG (pJD479, S3B). The control construct with just the changes in the 3′ portion of stem 3 was made from pJD465 (pJD567, S3A′). pJD465 was also used as the template for mutagenesis to move the A13456 residue out of stem 3 and into loop 2 (pJD492, S3C) and to make the change A13456C (pJD544, S3C′), while pJD467 was used to eliminate stem 3 entirely (pJD469, ΔS3). Stem 2 was also subjected to mutagenesis: the 5′ portion of stem 2 was changed from GCCCG to CGGGC (pJD466, S2A), and this in turn was the template for mutagenesis to make the complementary sequence in the 3′ half of stem 2, CAGGGC to GACCCG (pJD480, S2B). pJD465 was used as the template to create a construct in which the bulged A13467 residue in stem 2 was eliminated by moving it 6 nt downstream (pJD491, S2C) or replaced by cytosine (pJD542, S2C′).

**Table 1:**
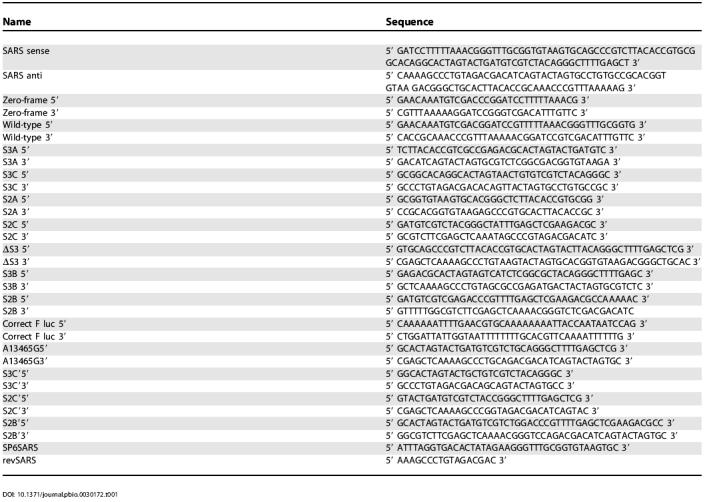
Oligonucleotides Used in This Study

An additional set of plasmids was constructed from the parental plasmids described above that lacked the yeast-specific markers but contained the SV40 early promoter, T7 promoter, and SV40 late poly (A) signal. These were used for programmed ribosomal frameshifting analyses in epithelial cells, wheat germ, and rabbit reticulocyte lysates. The BamHI and EcoRI fragment from pJD465 was purified and ligated into BamHI- and EcoRI-digested p2luc [18] to generate the test plasmid pJD502. A zero-frame control plasmid (pJD464) was constructed by cloning the BamHI/EcoRI fragment from pJD465 into p2luci. Similarly, BamHI and EcoRI fragments from the yeast plasmids described above were cloned into p2luc to generate a complete plasmid set for analyses of –1 PRF in epithelial cells (pJD503/S2A, pJD538/S2A′, pJD541/S2B′, pJD504/S2C, pJD537/S2C′, pJD487/S3A, pJD536/S3A′, pJD488/S3B, pJD506/S3C, pJD539/S3C′, and pJD490/ΔS3). An additional construct (A13465G) was made to control for the change at this position from adenine to guanine that prevents the creation of a termination codon in S2B′ constructs, but is not involved in stem 2 basepairing (pJD540 for Vero cells and pJD545 as the yeast plasmid).

#### Nuclease analysis.

The SP6SARS and revSARS oligonucleotides were used to generate a PCR amplicon from which an RNA transcript was made using the Ambion MEGAscript SP6 kit. The RNA was treated with calf intestinal phosphatase and 5′ end labeled with [γ-^32^P]ATP, using T4 polynucleotide kinase. The labeled RNA was gel purified and then eluted with 0.5 M NH_4_Ac, 1 mM EDTA, 0.1% SDS. Nuclease treatment with RNase A (1.0–0.01 ng), RNase T1 (1.0–0.01 U), and RNase V1 (0.1–0.001 U) from Ambion was performed according to the manufacturer's instructions for 15 min at room temperature. Digested RNA was electrophoresed through a 10% polyacrylamide gel and analyzed using a Storm PhosphoImager (Sunnyvale, California, United States).

#### Preparation of RNA samples for NMR**

A DNA construct (residues 13405–13472 of the SARS-CoV genome) was generated by PCR from pJD465 containing the wild-type SARS-CoV frameshift pseudoknot sequence. Two oligodeoxynucleotides (Invitrogen, Carlsbad, California, United States) were designed with a 5′ primer, including a T7 promoter sequence. The resulting PCR product was cloned into a pUC18 plasmid. To prepare milligram quantities of the SARS-CoV frameshift pseudoknot (residues 13405–13472), 7.5- to 20-ml in vitro transcription reactions with phage T7 polymerase from a linearized plasmid template were performed [48]. Unlabeled NTPs were purchased from Sigma Pharmaceuticals (South Croydon, United Kingdom), and labeled NTPs were purified from Methylophilus methylotropus ( ATCC 53528, American Type Culture Collection, Manassas, Virginia, United States) bacteria grown on labeled medium with ^15^N-ammonium sulfate and ^13^C-methanol [49]. After 4–5 h incubation at 37 °C, the reaction was spun down to remove traces of precipitated pyrophosphate. RNA transcripts were purified by anion-exchange FPLC with two HiTrap Q columns (Amersham Pharmacia, Piscataway, New Jersey, United States) equilibrated in 50 mM Tris (pH 8) at room temperature. The target RNA sample was eluted with an increasing sodium chloride gradient. Pure fractions were concentrated using a CentriPrep YM10 (Millipore, Billerica, Massachusetts, United States) concentrator, passed through a NAP25 column (Amersham Pharmacia) equilibrated with NMR buffer (20 mM potassium phosphate [pH 6.5], 200 mM potassium chloride, 0.5 mM EDTA [ethylene diamine tetraacetic acid disodium salt], 0.02% sodium azide, 5% deuterium oxide), and concentrated to 0.2–2 mM, using a CentriPrep YM10 concentrator (Millipore). The identity of the RNA product was verified by mass spectroscopy, as well as agarose and TBE-Urea-PAGE (Bio-Rad, Hercules, California, United States) gels.

#### NMR spectroscopy**

All NMR spectra were recorded at 5 °C, 15 °C, and 25 °C on a Bruker Avance 900 MHz spectrometer (Rheinstetten, Germany) equipped with a standard 5-mm triple axis pulsed field gradient ^1^H/^13^C/^15^N probehead optimized for proton detection. NMR experiments were performed on samples of 500-μl volume containing 0.2–2 mM SARS-CoV frameshift pseudoknot RNA. Data were processed using NMRPipe [50] and analyzed using NMRVIEW [51]. One-dimensional imino proton spectra were acquired using a jump-return echo sequence. The observable iminos in aqueous solution are diagnostic for hydrogen-bonded guanine and uracil bases, which are protected from exchange with the solvent. Imino resonances were assigned sequence-specificity from water flip-back, WATERGATE 2D nuclear Overhauser effect spectroscopy (NOESY) [52] spectra (τ_mix_ = 200 ms), and a jump-return [53] ^1^H,^15^N-heteronuclear multiple quantum correlation (HMQC) [54]. Elucidation of basepairing and secondary structure was verified from scalar ^2h^ *J*(N,N) couplings through hydrogen bonds in the quantitative *J*(N,N) HNN correlation spectroscopy (COSY) data [55,56].

## Supporting Information

Dataset S1 Molecular Genetic Analyses of Stems 2 and 3 The first page provides a summary of the final statistics for the frameshifting experiments shown in Figure 6. Subsequent pages show the raw data and subsequent analyses for all of the different constructs following the methodologies, as previously described [25].
(178 KB XLS).

### Accession Numbers

The GenBank (http://www.ncbi.nlm.nih.gov/Genbank/) accession numbers for the sequences discussed in this paper are AIBV (NC_001451), BCoV (NC_003045), HCoV-229E (NC_002645) , HCoV-HKU1 (NC_006577), HCoV-NL63 (NC_005831), HCoV-OC43 (NC_005147), MHV (NC_001846), PEDV (NC_003436), SARS-CoV (NC_004718), and TGV (NC_002306).

## Abbreviations

−1 PRF: programmed −1 ribosomal frameshift
2D: two-dimensional
MFE: minimum free energy
NMR: nuclear magnetic resonance
SARS: severe acute respiratory syndrome
